# Supplementary material for: Development of Machine-Learning-Based Facial Thermal Image Analysis for Dynamic Emotion Sensing
Source: Sensors (Basel). 2025 Aug 25;25(17):5276. doi: 10.3390/s25175276 (PMC12431138; doi:10.3390/s25175276)
Supplement: Supplementary file 1 [file sensors-25-05276-s001.zip › Supplementary Code S1.zip!/Supplementary Code 1/Alignment_project.zip!/Alignment_project/README.docx]

**Blender-Based Facial Nonlinear Alignment Guide**

**Introduction**

This project focuses on using Blender to perform nonlinear alignment of a face across video frames. The process involves manually aligning the mesh to the face in the first frame, followed by a script to automatically align the subsequent frames.

**Steps**

1. **Open the Project and Load the First Frame**
   - Open the Blender project file.
   - Import the first video frame as a background reference.
   - Ensure the image is clearly visible in the left image editor window.

2. **Manual Alignment of the First Frame**
   - **Select the mesh:** Use the left mouse button to select the mesh in the image.
   - **Move the mesh:** Press the G key to move the mesh to the desired position.
   - **Zoom the view:** Scroll the mouse wheel to zoom in or out.
   - **Pan the view:** Hold the mouse wheel to adjust the view position.
   - **Scale/Rotate the mesh:**
     - Use the Scale tool on the left toolbar to resize the mesh.
     - Use the rotation tool to rotate the mesh for better alignment.
   - **Hide/Show the mesh:** Press the TAB key to toggle the visibility of the mesh.
   - Adjust the mesh sections carefully until it closely matches the face.

3. **Check Shading and Render the Results**
   - Go to the shading tab at the top of the interface.
   - In the shading panel, load the same first frame image for preview.
   - Click "Render Image" from the top-left menu to view the alignment result.
   - If the alignment looks good, proceed to the next step.

4. **Set Up the Auto-Alignment Script**
   - Open the script editor in Blender.
   - Load the render script.
   - Update the following parameters in the script:
     - image_dir: Set this to the directory containing the video frame images.
     - points_file: Set this to the directory containing the facial landmark detection data.
     - base_render_path: Set this to the directory where the output files should be saved.
   - Verify the parameters are correct, then run the script.
5. **Run Automatic Alignment for Subsequent Frames**
   - The script will process and align all remaining frames automatically.
   - After the script completes, check the output directory for the alignment results.
