# Supplementary material for: Development of Machine-Learning-Based Facial Thermal Image Analysis for Dynamic Emotion Sensing
Source: Sensors (Basel). 2025 Aug 25;25(17):5276. doi: 10.3390/s25175276 (PMC12431138; doi:10.3390/s25175276)
Supplement: Supplementary file 1 [file sensors-25-05276-s001.zip › Supplementary Code S1.zip!/Supplementary Code 1/Landmark_detection.zip!/Landmark_detection/README.docx]

**README for Point Tracking Script in MATLAB**

**Environment**

This script is designed to run in MATLAB.

**Instructions**

1. **Set the Input and Output Paths**
   - Open the script in MATLAB.
   - Modify the following variables:
     - videoFile: Set this to the full path of the video you want to track.
       Example: 'E:\path_to_video\example_video.wmv'
     - outputFile: Set this to the full path of the CSV file where the tracking results will be saved.
       Example: 'C:\path_to_output\output_file.csv'
     - numPoints: Specify the number of points you want to track (typically, 2 points are sufficient).
       Example: numPoints = 2
2. **Run the Script**
   - Execute the script in MATLAB.
   - The first frame of the video will be displayed in a pop-up window.
3. **Mark the Points to Be Tracked**
   - Use the mouse to manually select the points on the first frame that you wish to track.
     Example: Two points between the eyebrows.
   - Once all the points are selected (equal to the number specified in numPoints), press the **Enter** key to start tracking.
4. **Tracking Process**
   - The script will track the selected points across all frames in the video.
   - The coordinates of the tracked points will be normalized and adjusted to match the Blender software's requirements:
     - **Normalization**: The coordinates are scaled by the frame dimensions.
     - **Y-coordinate inversion**: The Y-axis is flipped.
   - The adjusted coordinates are stored and saved in the specified output CSV file.
5. **Output Format**
   - The output CSV file will contain the following columns:
     - Frame: Frame number of the video.
     - U_A, V_A, U_B, V_B, ...: Normalized and inverted coordinates of the tracked points.
6. **Notes**
   - **Blender Compatibility**: This script is designed to output normalized and inverted coordinates that are compatible with Blender. If you intend to use the tracking data for other purposes, modify the following section of the code. Remove or adjust the normalizedPoints logic to fit your requirements.

- **Head Movement**: If the participant's head movements are too large, the tracking points may be lost. In such cases, select different points and re-run the script.
