# Supplementary material for: Development of Machine-Learning-Based Facial Thermal Image Analysis for Dynamic Emotion Sensing
Source: Sensors (Basel). 2025 Aug 25;25(17):5276. doi: 10.3390/s25175276 (PMC12431138; doi:10.3390/s25175276)
Supplement: Supplementary file 1 [file sensors-25-05276-s001.zip › Supplementary Code S1.zip!/Supplementary Code 1/ML_model.zip!/ML_model/README.docx]

**ResNet Model**

This is a pretrained ResNet-34 model for predicting arousal values from thermal facial images. The model is trained for regression tasks and outputs a single continuous value representing the predicted arousal level.

**Input Format**

- **Input Type**: Thermal facial images (RGB or single-channel heatmaps).
- **Input Size**: 224 × 224 pixels.

**Output Format**

- **Output Type**: A single continuous value (arousal).
- **Range**: The predicted arousal value depends on the dataset used during training. Ensure you normalize your ground truth accordingly if necessary.

**Linear Regression Model Fit**

The linear regression model produced a fitted curve with the equation:

y=0.9483x+0.3416

This equation is derived from the following code:

slope, intercept = np.polyfit(y_test, y_pred, 1)

print(f"Fitted Line: y = {slope:.4f}x + {intercept:.4f}")
